# Preparation and Characterization of Novel Green Seaweed Films from *Ulva rigida*

**DOI:** 10.3390/polym15163342

**Published:** 2023-08-08

**Authors:** Uruchaya Sonchaeng, Phanwipa Wongphan, Wanida Pan-utai, Yupadee Paopun, Wiratchanee Kansandee, Prajongwate Satmalee, Montakan Tamtin, Prapat Kosawatpat, Nathdanai Harnkarnsujarit

**Affiliations:** 1 Department of Packaging and Materials Technology, Faculty of Agro-Industry, Kasetsart University, Bangkok 10900, Thailand; 2 Department of Applied Microbiology, Institute of Food Research and Product Development, Kasetsart University, Bangkok 10900, Thailand; 3 Scientific Equipment and Research Division, Kasetsart University Research and Development Institute, Kasetsart University, Bangkok 10900, Thailandrdiwnk@ku.ac.th (W.K.); 4 Department of Food Chemistry and Physics, Institute of Food Research and Product Development, Kasetsart University, Bangkok 10900, Thailand; ifrpws@ku.ac.th; 5 Kung Krabaen Bay Royal Development Study Center, Department of Fisheries, Ministry of Agriculture and Cooperatives, Chantha Buri 22120, Thailand; 6 Phetchaburi Coastal Aquaculture Research and Development Center, Coastal Aquaculture Research and Development Division, Department of Fisheries, Ministry of Agriculture and Cooperatives, Phetchaburi 76100, Thailand; prapat1120@gmail.com; 7 Center for Advanced Studies for Agriculture and Food, Kasetsart University, Bangkok 10900, Thailand

**Keywords:** algae, biomaterial, biopolymer, bioplastic, food packaging, edible film

## Abstract

*Ulva rigida* green seaweed is an abundant biomass consisting of polysaccharides and protein mixtures and a potential bioresource for bioplastic food packaging. This research prepared and characterized novel biodegradable films from *Ulva rigida* extracts. The water-soluble fraction of *Ulva rigida* was extracted and prepared into bioplastic films. ^1^H nuclear magnetic resonance indicated the presence of rhamnose, glucuronic and sulfate polysaccharides, while major amino acid components determined via high-performance liquid chromatography (HPLC) were aspartic acid, glutamic acid, alanine and glycine. Seaweed extracts were formulated with glycerol and triethyl citrate (20% and 30%) and prepared into films. *Ulva rigida* films showed non-homogeneous microstructures, as determined via scanning electron microscopy, due to immiscible crystalline component mixtures. X-ray diffraction also indicated modified crystalline morphology due to different plasticizers, while infrared spectra suggested interaction between plasticizers and *Ulva rigida* polymers via hydrogen bonding. The addition of glycerol decreased the glass transition temperature of the films from −36 °C for control films to −62 °C for films with 30% glycerol, indicating better plasticization. Water vapor and oxygen permeability were retained at up to 20% plasticizer content, and further addition of plasticizers increased the water permeability up to 6.5 g·mm/m^2^·day·KPa, while oxygen permeability decreased below 20 mL·mm/m^2^·day·atm when blending plasticizers at 30%. Adding glycerol efficiently improved tensile stress and strain by up to 4- and 3-fold, respectively. Glycerol-plasticized *Ulva rigida* extract films were produced as novel bio-based materials that supported sustainable food packaging.

## 1. Introduction

Green seaweeds, widely distributed in coastal areas, are rich sources of polysaccharides and amino acids. *Chlorophyceae* seaweeds from the *Ulva* genus are world renowned because of the bloom phenomenon ‘green tides’ that negatively impacts tourism areas and marine life [1,2]. Currently, *Ulva* biomass is harvested to produce compost, fertilizers and biofuels. The biomass has good nutritional qualities and contains proteins, dietary fiber and complex polysaccharide components. Previous investigations indicated that *Ulva* seaweeds contain cellulose and hemicellulose as major components, with the potential to form networks and utilization as sustainable packaging [1,2,3,4,5,6,7]. However, these cellulosic materials are rarely soluble in water, suggesting the formation of film networks from a non-water-soluble fraction. Conversely, the present study demonstrated the film formation from the water-soluble fraction of *Ulva rigida*. The development of *Ulva* materials into biodegradable films and packaging would support sustainable development goals while increasing the value of this abundant biomaterial.

Biopolymers are attractive alternative materials to replace conventional petroleum-based packaging films which cause severe environmental problems. The accumulation of non-biodegradable plastic waste leads to pollution in landfills. Developments using biopolymer films including chitosan, alginate and cellulose derivatives have recently attracted attention [6,8,9,10,11]. However, biopolymers have limited mechanical and barrier properties. Plasticizers can be incorporated into film components to improve mechanical properties and flexibility [12,13].

New sources of biomaterials are now increasingly investigated to seek alternative renewable resources for packaging materials. Novel polymeric packaging from seaweeds has been investigated and developed, e.g., *Laminaria japonica* and *Sargassum natans* [8], *Alaria esculenta* and *Saccharina latissimi* [10] and *Furcellaria lumbricalis* and *Gigartina skottsbergii* seaweeds [9]. Seaweeds contain mixtures of organic (e.g., carbohydrates, proteins and fibers) and inorganic substances (e.g., sodium, potassium and calcium) with polysaccharide composition of cellulose, alginate (anionic polysaccharides) and several sulfated polysaccharides as the main cell wall components. Sulfated polysaccharides are commonly characterized by the presence of sulfate (${\mathrm{SO}}_{4}^{2-})$ functional groups, named ‘ulvans’ in green seaweeds. These component mixtures are soluble and insoluble in different solvents [3]. Insoluble substances form phase separation due to their immiscibility, which causes non-homogeneous film materials and results in poor mechanical strength and high permeability of volatile compounds [12,14]. Water and solvent extraction of seaweed components leads to similar properties of the extracts that are suitable for conversion into polymeric films to achieve more homogeneous structures. *Ulva rigida* contains high amounts of cell wall polysaccharides including cellulose and water-soluble polysaccharides containing mainly ulvan sulfate groups. The major component is a disaccharide formed by β-d-glucuronic acid (1,4)-l-rhamnose-3-sulfate [3]. Mixed hydrocolloid components make *Ulva rigida* a potential resource for film and packaging materials, but detailed studies are lacking. The formation of *Ulva* biopolymer films requires the addition of plasticizers to improve deformability and stretchability.

This research prepared and characterized biopolymer films from *Ulva rigida* crude extracts. Films were formulated with different plasticizers (glycerol and triethyl citrate). The morphology, chemical structures, mechanical and barrier properties of the films were determined. Findings support the utilization of *Ulva rigida* as a novel renewable resource for biodegradable packaging to enhance the value of sustainable materials.

## 2. Materials and Methods

### 2.1. Preparation and Extraction of Ulva rigida

**Figure 1:**
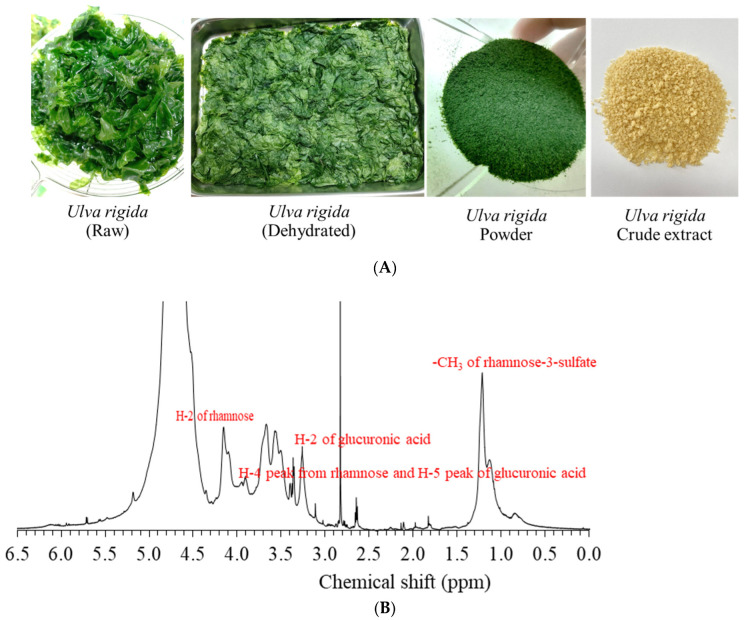
(**A**) Appearance of fresh, dehydrated, powdered and extract of *Ulva rigida*; (**B**) The ^1^H NMR spectrum of *Ulva rigida* extract in D_2_O.

*Ulva rigida* (donated from the Phetchaburi Coastal Aquaculture Research and Development Center, Department of Fisheries, Phetchaburi, Thailand) was collected after 21 days of cultivation in seawater at salinity 30–32 ppm (Figure 1A). Fresh *Ulva rigida* was harvested and washed before drying in a hot air oven at 60 °C for 3–6 h. Dried biomass was milled, giving particle size of 0.5 mm. Water-soluble substances were extracted according to Hamouda et al. [7] with minor modifications. The oven-dried biomass was added with distilled water at a biomass/solvent ratio of 1:20 (%*w*/*v*) under controlled temperature of 90 °C and extraction time of 120 min. The supernatant was separated by high-speed refrigerated centrifugation (Model 6000, Kubota, Tokyo, Japan) at 10,000× *g* for 10 min at 25 °C. An amount of 3 mL of ethanol was added to the supernatant before storing at 4 °C for 24 h. The precipitate was recovered and dried overnight at 50 °C.

### 2.2. Analysis of the Biochemical Composition of Ulva rigida Extracts

Oven-dried *Ulva rigida* biomass was analyzed to determine the biochemical composition following AOAC methods [15]. Moisture content was determined via oven-drying at 105 °C to constant weight. Ash content was determined via ignition of the dried samples in an electric furnace at 550 °C. Protein content was determined via the Kjeldahl method using a nitrogen conversion factor of 6.25. Total lipid content was determined using a modified Bligh and Dyer method [16]. Briefly, the samples were suspended in distilled water, methanol and chloroform at a ratio of 0.8:2.0:1.0 and mixed well. The mixture was ultrasonically homogenized for 15 min and then separated via centrifuging at 6000× *g* rpm for 15 min. The lipid phase was collected, and the cell debris was extracted until the cells had no color. The lipid extract was then filtered to remove contaminated cell debris and dried to constant weight at 80 °C.

Amino acids in the *Ulva rigida* biomass were extracted and quantified according to the method of Al-Dhabi and Valan Arasu [17] with slight modifications. Briefly, 100 mg of biomass was mixed with 5 mL of 6 N HCl. The slurry sample mixture was incubated at 110 °C for 24 h and then cooled. The sample was then diluted to pH 2.2 with 10 M NaOH and filtered through a 0.45 PTFE syringe filter. The filtrate was determined via high-performance liquid chromatography (HPLC) with a fluorescent detector (HP 1260, Agilent Technologies, Waldbronn, Germany) using superficially porous particles (SPP) technology 4.6 × 100 mm AdvanceBio AAA (2.7 μm) column (Agilent). The oven temperature of the column was set at 30 °C. The sample was derivatized with o-phthalaldehyde (OPA) and 3-mercaptopropionic acid and injected into the column. The gradient mobile phase consisted of a mixture of solvent A (40 mM NaH_2_PO_4_) and solvent B (methanol, acetonitrile and water). Flow rate was set at 0.7 mL min^−1^, and peaks were measured as Ex/Em at 340/450 nm. Amino acid standard solution (AA-S-18, Sigma-Aldrich, Singapore) was used as the external standard to calculate the amino acid composition.

### 2.3. ^1^H Nuclear Magnetic Resonance (NMR) of Ulva rigida Extracts

The ^1^H NMR spectra were carried out on Ascend^TM^ 600/Avance III HD (Bruker, Billerica, MA, USA). Sample powder (16 mg) was dissolved in D_2_O. The solution was operated at 600 MHz running TopSpin 3.6.4 software (Bruker).

### 2.4. Preparation of Ulva rigida Films

*Ulva* films were prepared by dissolving 1.5% (*w*/*w*) of crude polysaccharides into distilled water. The formulations of the suspensions were prepared using different plasticizers, namely glycerol (Asian Scientific Co., Ltd., Samutprakarn, Thailand), triethyl citrate (pure Ph. Eur., NF, PanReac AppliChem ITW Reagents, Darmstadt, Germany), or a combination of glycerol and triethyl citrate in a 1:1 ratio. Concentrations of plasticizer at 0%, 20% and 30% (*w*/*w* of crude polysaccharide weight) were investigated. The suspensions were prepared into films via solution casting with continuous stirring at room temperature (25 ± 3 °C) for 3 h using a magnetic stirrer (IKA Magnetic Stirrers C-MAG HS 7, IKA^®^ Works (Thailand) Co., Ltd., Bangkok, Thailand). Air bubbles were removed via an ultrasonic bath (Sonorex Digitec DT 255 H-RC, Bandelin Electronic GmbH & Co. KG, Berlin, Germany) for 30 min. Blend suspensions (50 ± 5 g) were poured onto polystyrene Petri dishes (diameter 140 mm) and dried at 50 °C for 15 h in a hot air oven. The dried films were removed from the plate and stored in a temperature–humidity-controlled chamber (Climate Chamber Binder KBF 720, Binder GmbH, Tuttlingen, Germany) at 50% relative humidity before analyses for at least 48 h. Thickness of films was determined using a micrometer (model ID-C112BS, Mitutoyo, Kanagawa, Japan).

### 2.5. Morphology of Ulva rigida Films

#### 2.5.1. Microstructure

The surface and cross-section morphology of the films was observed by a FEI Quanta 450 Scanning Electron Microscope (SEM) (Thermo Fisher Scientific, Waltham, MA, USA) at 15 kV and magnification of 500× and 3000×, respectively. Film samples were immersed and cracked in liquid nitrogen, and specimens were covered with gold using a sputter coater (Quorum Technology Polaron Range SC7620, East Sussex, UK) to facilitate electrical conductivity.

#### 2.5.2. X-ray Diffraction Analysis

Crystallinity was determined using X-ray diffraction (Diffractometer D8, Bruker AXS, Karlsruhe, Germany) at a scanning rate of 0.8/s and a 0.02° step size. The scanned region ranged from 4° to 40° using voltage and current of 40 kV voltage and 40 mA, respectively.

### 2.6. Fourier Transform Infrared Spectroscopy (FTIR) of Ulva rigida Films

FTIR spectra of the film samples were recorded using a Model 400 Fourier transform infrared spectrometer (Perkin Elmer, Beaconsfield, UK) with attenuated total reflectance (ATR) mode. Absorbance spectra were obtained at 500–4000 cm^−1^ wavelength with anvil geometry of 45° at 4 cm^−1^ resolution and 64 scanning times. The spectra were standardized with the spectrum of air. Test was carried out in eight replications.

### 2.7. Thermal Stability and Properties of Ulva rigida Films

#### 2.7.1. Thermogravimetric Analysis (TGA)

Thermal stability of the films was determined via thermogravimetric analysis (TGA 2 STARe System, Mettler Toledo, Greifensee, Switzerland). Film pieces (10–15 mg) were placed in aluminum pans, sealed and scanned over the range 25–900 °C under a nitrogen atmosphere at flow rate of 20 mL/min with heating rate of 10 °C/min. First derivative graphs were derived from the weight loss values of the samples.

#### 2.7.2. Differential Scanning Calorimetry (DSC)

Thermal properties of the films were determined using a differential scanning calorimeter (DSC 1, STARe System, Mettler Toledo, Greifensee, Switzerland). Films (1–2 mg) were placed in aluminum pans under nitrogen flux with a flow rate of 50 mL/min. The films were heated from 25 to 80 °C with a heating rate of 10 °C/min to remove moisture in the sample, followed by cooling from 80 to −80 °C at a cooling rate of 10 °C/min. Finally, the films were heated to 300 °C at a rate of 10 °C/min.

### 2.8. Surface and Barrier Properties of Ulva rigida Films

#### 2.8.1. Water Contact Angle

Hydrophobicity of the film surface was determined via contact angle measurement (Dataphysics OCA 15EC, Dataphysics Instruments GmbH, Filderstadt, Germany). A 3 μL droplet of distilled water was dropped on the film surface using a microsyringe connected to a computer system. Images were immediately taken, and contact angle values were averaged from nine samples using the SCA 20 software version 2 (Dataphysics). Data were averaged from at least 5 samples.

#### 2.8.2. Water Vapor Permeability (WVP)

Water vapor transmission rate (WVTR) was tested using the standard cup method following ASTM E96-80. The samples were kept at 25 ± 2 °C and 50 ± 2% RH in a humidity chamber (Binder KBF 720, Binder GmbH, Tuttlingen, Germany). Triplicate film samples were cut into a circle (7 cm diameter), placed on a metal cup containing silica gel and sealed with an O-ring using paraffin wax. The cups were weighed periodically until constant weight. Water vapor permeability was calculated from triplicate samples using Equation (1).

WVP = (WVTR × thickness)/ΔP(1)

where WVTR is derived from the slope of the linear regression line obtained from plotting the weight gain against time, and ΔP is the vapor pressure difference.

#### 2.8.3. Oxygen Permeability (OP)

Oxygen permeability was calculated from the oxygen transmission rate (OTR) determined using an oxygen permeation analyzer (Model 8500, Illinois Instruments, Johnsburg, IL, USA) according to ASTM D3985-81. The OP was calculated from duplicate samples using Equation (2).

OP = (OTR × thickness)/ΔP(2)

where ΔP is the oxygen partial pressure across the film.

### 2.9. Mechanical Properties of Ulva rigida Films

Mechanical properties were determined using an Instron Universal Testing Machine (Model 5965, Instron, Norwood, MA, USA) according to ASTM D882-88. The samples were cut into rectangular pieces (150 mm × 25 mm) and tested in duplicate at a speed of 500 mm/min. Distance between the gap was 5 cm. Results were plotted between tensile stress (MPa) and tensile strain (%).

### 2.10. Statistical Analysis

Statistical analyses were conducted to determine significant differences among film sample data via analysis of variance (ANOVA) using IBM SPSS Statistics version 22.0 (IBM Corp., Armonk, NY, USA) and Duncan’s multiple range test, with significance set at *p* < 0.05.

## 3. Results and Discussion

### 3.1. Characterization of Ulva rigida Seaweed Extracts

#### 3.1.1. Proximate Analysis and Amino Acid Composition

Proximate analysis results showed that *Ulva rigida* seaweed extracts consisted of 22.42 ± 0.72% carbohydrate, 19.01 ± 0.92% protein, 5.67 ± 0.44% lipid, 5.53 ± 0.20% crude fiber and 40.21 ± 0.90% ash. The amino acid profile of *Ulva rigida* extract is shown in Table 1. Major amino acid components consisted of aspartic acid, glutamic acid, alanine and glycine. Seaweed is a source of polysaccharides with protein (10 to 27%) and minor contents of lipid (0.2–3%) [2,10]. Shuuluka et al. [5] indicated major amino acid components of *Ulva rigida* as aspartic acid, alanine and glutamic acid (13.0  ±  1.1, 12.3  ±  0.7 and 9.4  ±  1.0 g/100 g protein, respectively). Similarly, Brain-Isasi et al. [2] reported levels of glutamic acid, aspartic acid and alanine as 16.31 ± 0.82, 14.56 ± 0.73 and 9.89 ± 0.51 g/100 g protein, respectively, as the major amino acid components. Differences in amino acid components depend on several factors including season and environment [2,5]. These amino acids are small molecular weight substances that plasticize the film matrix and improve deformability and flexibility.

#### 3.1.2. Nuclear Magnetic Resonance (NMR)

The ^1^H NMR spectrum of *Ulva rigida* extract in D_2_O is shown in Figure 1B. A strong ^1^H resonance at 1.22 ppm, corresponding to the methyl groups of rhamnose-3-sulfate [4]. Kidgell et al. [1] also reported similar proton resonance peaks in ulvans at 3.3 ppm and 3.7 ppm attributed to H-2 and H-3/H-4 of glucuronic acid, respectively, while peaks at 3.8–3.9 ppm were due to combination of the H-4 peak from rhamnose and H-5 peak of glucuronic acid. The major ^1^H NMR peak at 4.2 ppm was from H-2 of rhamnose.

### 3.2. Film Microstructure

**Figure 2:**
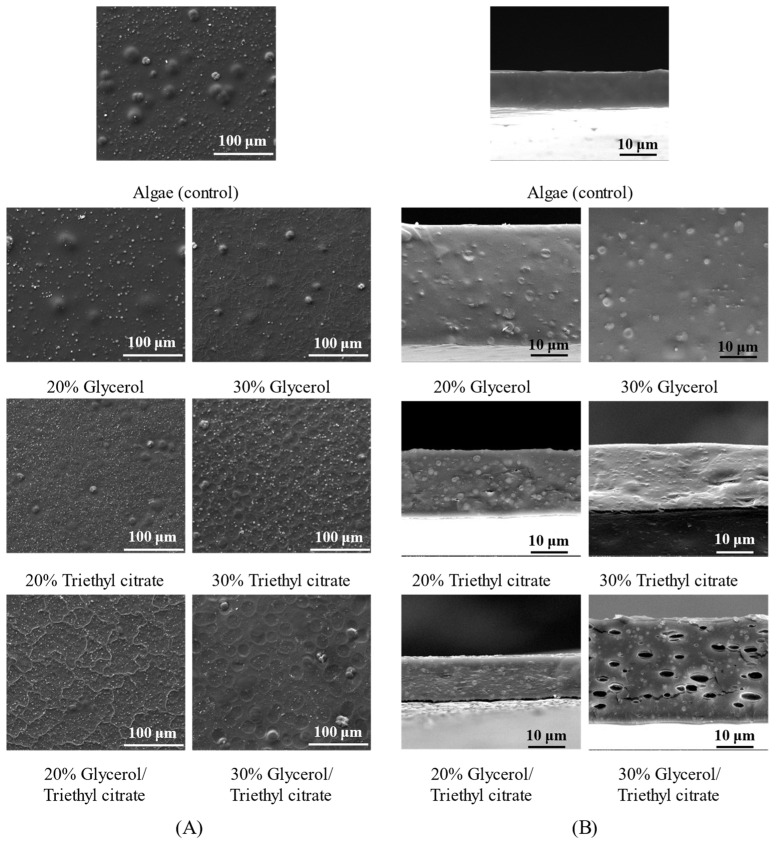
Microstructures as (**A**) surface and (**B**) cross-section of *Ulva rigida* films containing glycerol, triethyl citrate and their blends (glycerol/triethyl citrate) at 20% and 30%.

Surface microstructures of the films are shown in Figure 2A. All films showed numerous finely dispersed particles spreading on the surface. Triethyl citrate at both concentrations showed greatly increased amounts of fine particles. Adding glycerol reduced the number of fine particles. Some larger clumps embedded beneath the film surface with size ranging from 15 to 40 μm. The control film showed higher numbers of large clumps. Figure 2B shows cross-section images of the matrices as fine particles less than 10 μm. Triethyl citrate-plasticized films contained the highest numbers of fine particles. The non-homogeneity of the film matrices reflected the immiscibility of film components. No cracks were found in all films including the control (Figure 2A,B) suggesting strong bonding of the polymeric networks [8]. The presence of protein in *Ulva rigida* enhanced the bonding and strength of the polymer networks via several interactions including electrostatic interactions, hydrophobic interactions and hydrogen bonds [10,18]. Some of these components formed aggregates due to favorable thermodynamics, while the recrystallization of polymer components caused fine crystallites. Accordingly, the phase separation of aggregates and crystallites occurred, causing non-homogeneous matrices.

### 3.3. Fourier Transform Infrared Spectroscopy

**Figure 3:**
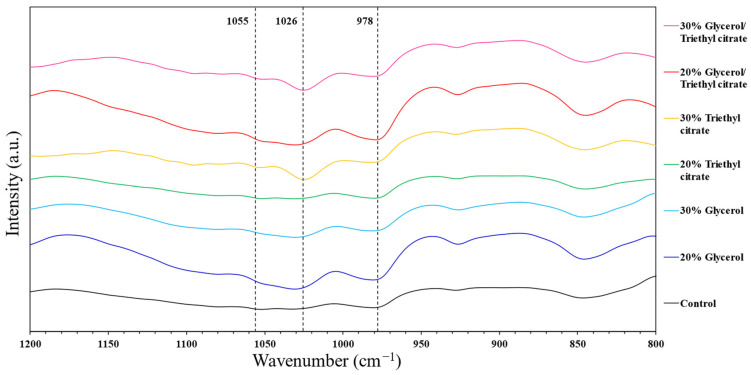
FTIR spectra of *Ulva rigida* films containing glycerol, triethyl citrate and their blends (glycerol/triethyl citrate) at 20% and 30%.

Infrared (IR) absorption spectra of the films are shown in Figure 3. The absorption peaks and corresponding functional groups are shown in Supplementary Table S1. The control film had maximum absorption peaks at 1026 cm^−1^ and 1055 cm^−1^, ascribed to C-O stretching vibrations in C-O-C that were sensitive to the amounts of disorder amorphous and order crystalline structures of the polymers, respectively [19,20]. The addition of both plasticizers (both glycerol and triethyl citrate) increased the intensity of the absorption peak at 1026 cm^−1^ and merged the peak at 1055 cm^−1^, reflecting the amorphous structures of the plasticizers. The absorption peaks at 980 and 848 cm^−1^ were assigned to asymmetrical and symmetrical stretching vibration of the C-O-S bond due to the presence of sulfate polysaccharides, respectively [21].

Glycerol had similar IR absorption spectra to the control film in the fingerprint region (500–1500 cm^−1^) due to similar vibration of C-H bending (1500–1200 cm^−1^) in CH_2_ and C-O stretching (980–1250 cm^−1^). Higher concentrations of triethyl citrate increased absorption at 1735 cm^−1^ attributed to C = O stretching vibration of ester bonds in the triethyl citrate structure [12]. The band at 1425 cm^−1^ was associated with vibration of C-OH deformation in O–C–O symmetric stretching vibration of the carboxylate group in seaweed polymers [10]. The IR wide absorption band at 1629 cm^−1^ was due to carboxyl groups in *Ulva* spp. which overlapped with C = C stretching vibration of the methacrylic group [4]. The IR spectra showed peaks around 1200–1250 cm^−1^ and 840–845 cm^−1^, ascribed to S = O stretching and C–O–S stretching characteristics of polysaccharides in *Ulva* spp. [6]. The wide absorption peak between 3000 and 3700 cm^−1^ was ascribed to O-H stretching vibration, and attributed to intra- and inter-molecular hydrogen bonding. Small absorption peaks between 2850 and 3050 cm^−1^ were attributed to C-H stretching vibration [10,20,22] and were stronger in films containing plasticizers. Adding glycerol intensified the spectrum at 3240 cm^−1^, suggesting hydrogen bonding between the polymer and glycerol, while adding triethyl citrate gave a wider peak with stronger absorption at 3495 cm^−1^, due to hydrogen bonding with polymer components. The results indicated interactions between plasticizers and polymers, which contributed to plasticization effects of the films.

### 3.4. Thermogravimetric Analysis

**Figure 4:**
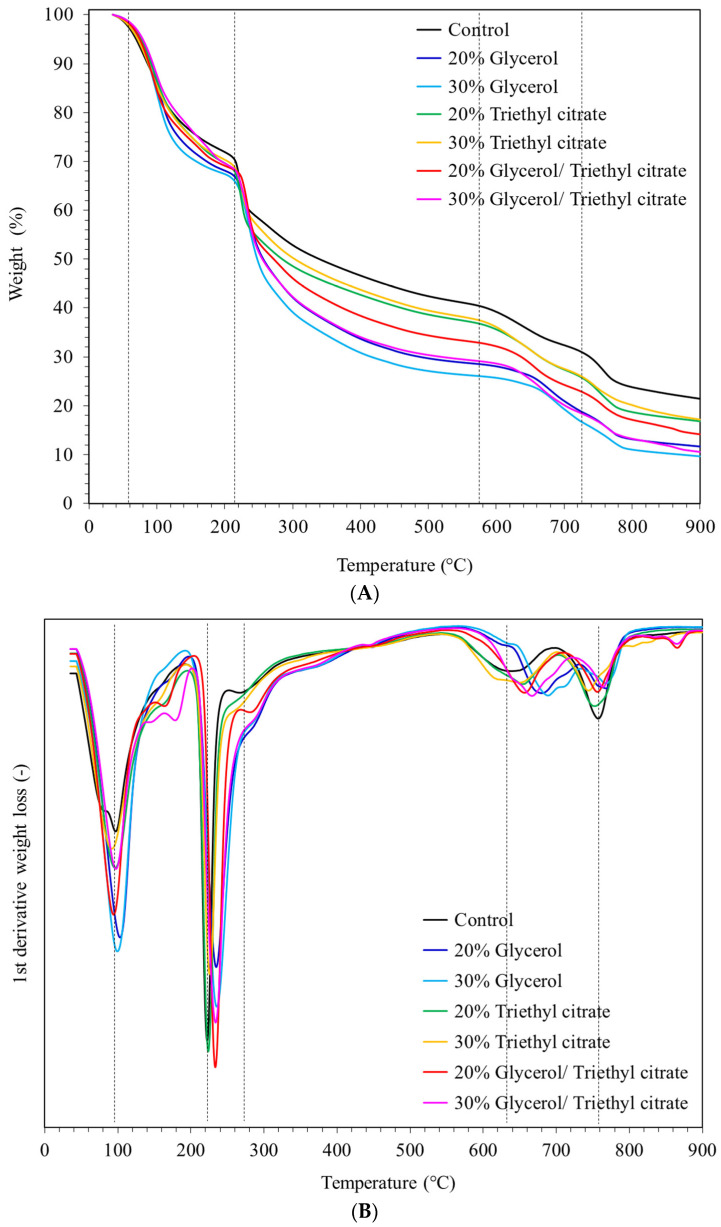
Thermal degradation as (**A**) weight percent and (**B**) first derivative weight loss of *Ulva rigida* films containing glycerol, triethyl citrate and their blends (glycerol/triethyl citrate) at 20% and 30%.

Figure 4A shows weight loss, reflecting thermal degradation and volatilization of film components. The onset temperature, weight loss in each stage of degradation and char residue is shown in Supplementary Table S2. The first sharp weight loss started at 60 °C and was due to evaporation of absorbed water, corresponding with mass loss of 20–28% depending on plasticizer types and concentrations. Glycerol-plasticized films had the highest mass loss, reflecting the highest levels of absorbed water attributed to high hydrophilicity from large numbers of hydroxyl groups. The second weight loss of the control film started at 215 °C, corresponding with an approximately 10% weight reduction followed by a sharp weight loss due to volatilization of polymer components [3,6,8,12]. Triethyl citrate-plasticized films had a similar weight reduction to the control, while films with glycerol showed a higher weight reduction, corresponding with the amounts of plasticizers and due to volatilization of glycerol [12,23]. The final residue left after the thermal decomposition process decreased with the addition of plasticizer, indicating the breaking of polymer–polymer interactions with the addition of glycerol and triethyl citrate.

The first derivative weight loss of the films is shown in Figure 4B. The control film showed a peak with a shoulder, suggesting evaporation of multi-components as free and bound water below 100 °C [12]. Films containing plasticizers showed sharper peaks at 100 °C due to water evaporation, suggesting higher levels of water absorption. Mixtures of plasticizers showed two extra volatilization peaks between 135 and 200 °C that did not occur in glycerol- and triethyl citrate-plasticized films. Mixtures of the plasticizers stabilized the film components, giving higher degradation temperatures. The sharpest degradation started at 200 °C in all films. The control and triethyl citrate-plasticized films had a major degradation peak at 222 °C, while glycerol-plasticized films showed a sharp peak at 235 °C. Shifting of the degradation peak at around 230 °C in the blend plasticizers reflected the major role of glycerol on the degradation temperature in protein and polysaccharide components [6,10,23]. A small shoulder was found at 265 °C for the control and triethyl citrate films, and at 285 °C for the films with glycerol and the mixtures. Degradation at around 300 °C was due to polymer components. These findings reflected the interactions between glycerol and the polymers, which had a major effect on thermal stability.

### 3.5. Differential Scanning Calorimetry (DSC)

**Figure 5:**
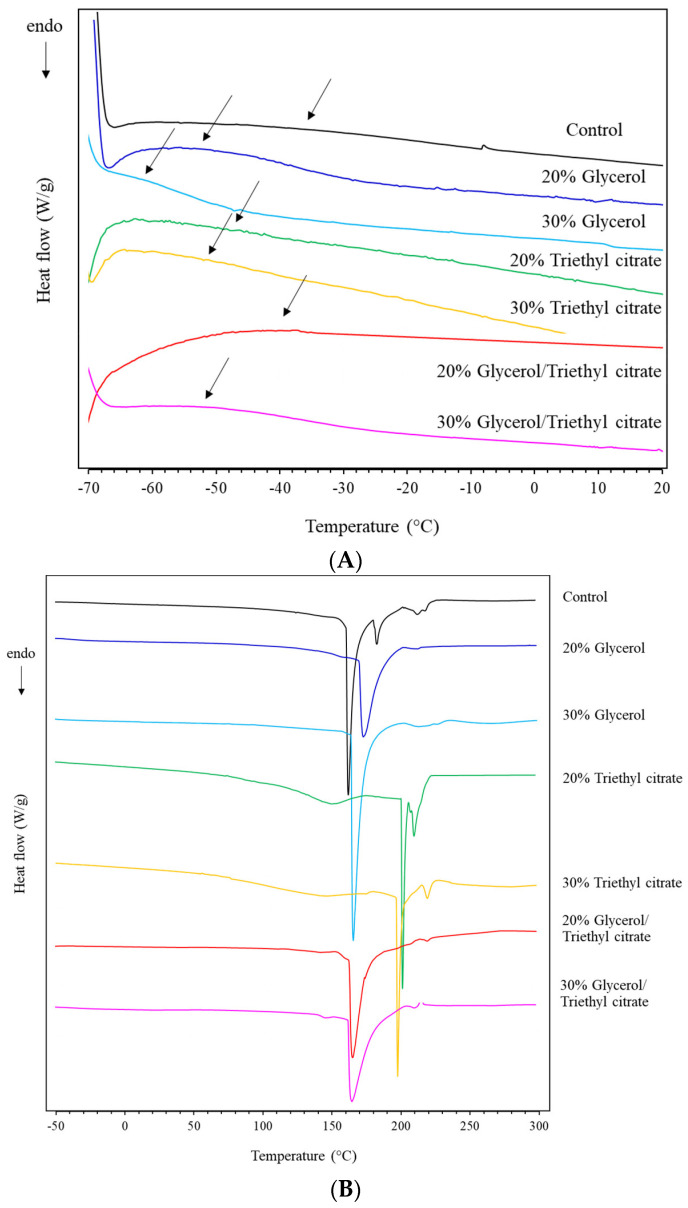
DSC thermograms (**A**) enlarged at low temperature range and (**B**) between −50 °C and 300 °C in the second scan of *Ulva rigida* films containing glycerol, triethyl citrate and their blends (glycerol/triethyl citrate) at 20% and 30%. The arrows indicate endothermic shift, suggesting the glass transition temperature (T_g_) of the films.

The DSC thermograms showed an endothermic shift in heat flow, suggesting the glass transition temperature (T_g_) of the films (Figure 5A). The control film had onset T_g_ at −36 °C. *Ulva rigida* extract consisted of small molecular weight solids, particularly amino acids (Table 1). These small molecules plasticized the polysaccharide film matrices resulting in low T_g_ values. The addition of both plasticizers (glycerol and triethyl citrate) and their mixtures reduced the T_g_ of the films. Glycerol decreased T_g_ values more than triethyl citrate, corresponding with T_g_ values of −53 °C and −62 °C for films containing 20% and 30% glycerol, respectively. Increasing plasticizer contents gave a lower T_g_ due to higher plasticization effects, with a larger magnitude of endothermic shift found in glycerol-plasticized films. These results reflected stronger plasticization effects of glycerol in seaweed extract films. Glycerol has an extremely low T_g_ value of −86 °C [24], while the molecular weight of triethyl citrate is higher than glycerol, corresponding with a lower mole number at the same weight. A smaller plasticizer size, namely glycerol, also enhanced the dispersion and interactions with the polymer in the matrices [13,25]. Glycerol molecules consist of three hydroxyl groups (-OH) per mole, which readily form hydrogen bonding with polysaccharides. Mixtures of glycerol and triethyl citrate gave intermediate T_g_ between their blends. The plasticizers decreased the T_g_ by disrupting the intermolecular chain movement of the polymers, thereby improving the segmental mobility of the polymers. A hydrophilic plasticizer such as glycerol facilitates more interactions between itself and the polymer, resulting in an increase in the free volume of matrix, thereby reducing the T_g_. Hence, the results clearly indicated interaction between glycerol and triethyl citrate with the polymer matrices causing T_g_ reduction.

Figure 5B shows endothermic peaks, reflecting phase transitions of film components starting above 70 °C due to water evaporation. The sharp endothermic peaks between 150 and 220 °C reflected the melting of the crystallites, as discussed in the X-ray diffractions results. The control film showed a sharp peak at 160 °C followed by smaller peaks at 183 °C and 212 °C. Glycerol-plasticized films showed a single but wider peak than the control at higher temperature. Triethyl citrate showed a wide peak between 75 and 175 °C, followed by a sharp peak around 200 °C and a smaller peak at higher temperatures (similarly to the control). The results suggested that triethyl citrate enhanced the non-homogeneous crystallization of film components, corresponding with wide and multiple melting temperatures.

### 3.6. X-ray Diffraction

**Figure 6:**
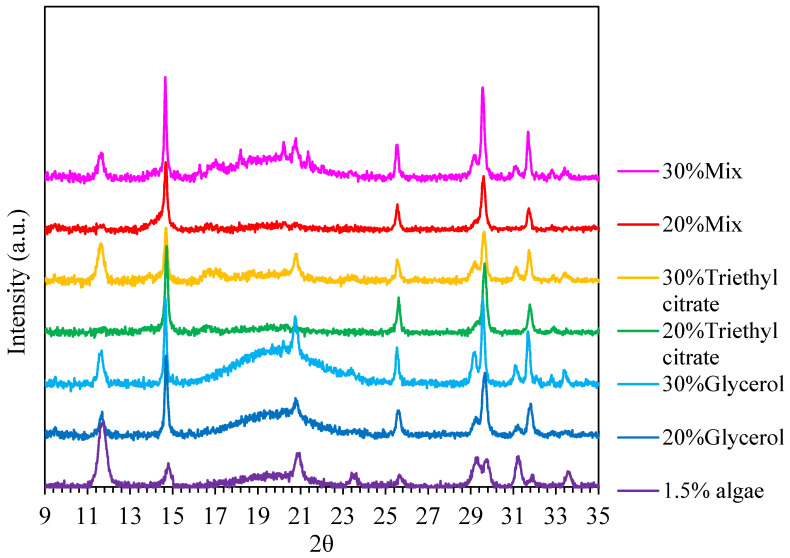
X-ray diffractograms of *Ulva rigida* films containing glycerol, triethyl citrate and their blends (glycerol/triethyl citrate) at 20% and 30%.

X-ray diffractograms of films with different plasticizers are shown in Figure 6. The films had diffraction angles at 2θ = 11.7, 14.6, 20.8, 22.5, 25.5, 29.2, 29.6, 31.1 and 31.7°. Glycerol clearly caused a broad peak between diffraction angles in the range of 16–25°, reflecting amorphous components. Conversely, triethyl citrate gave a lower intensity of the diffraction curve, suggesting reduced amorphous components. The DSC analysis (Figure 5B) also indicated that triethyl citrate-plasticized films showed large and wide melting peaks, reflecting the higher levels of crystalline components. The sharp peak at 14.6° (corresponding to the (110) crystalline plane) was much more intense, suggesting a preferential orientation of the (110) crystal plane parallel to the film surface [9]. The intensity of the peaks at 2θ = 11.7, 20.8, 29.2, 31.3 and 33.7° decreased when adding 20% plasticizers (glycerol and triethyl citrate and their mixtures), while a further increase in plasticizers to 30% increased peak intensity of these aforementioned peaks. Adding plasticizers increased peak intensity at 2θ = 14.6, 25.5, 29.6 and 31.7°. The reduction in crystallinity was attributed to amorphous complexes formed through intermolecular interactions between film-forming substrates and plasticizers [11]. The results suggested that glycerol and triethyl citrate impacted the morphology of the crystalline structures in seaweed films.

### 3.7. Surface Hydrophobicity and Barrier Properties

**Figure 7:**
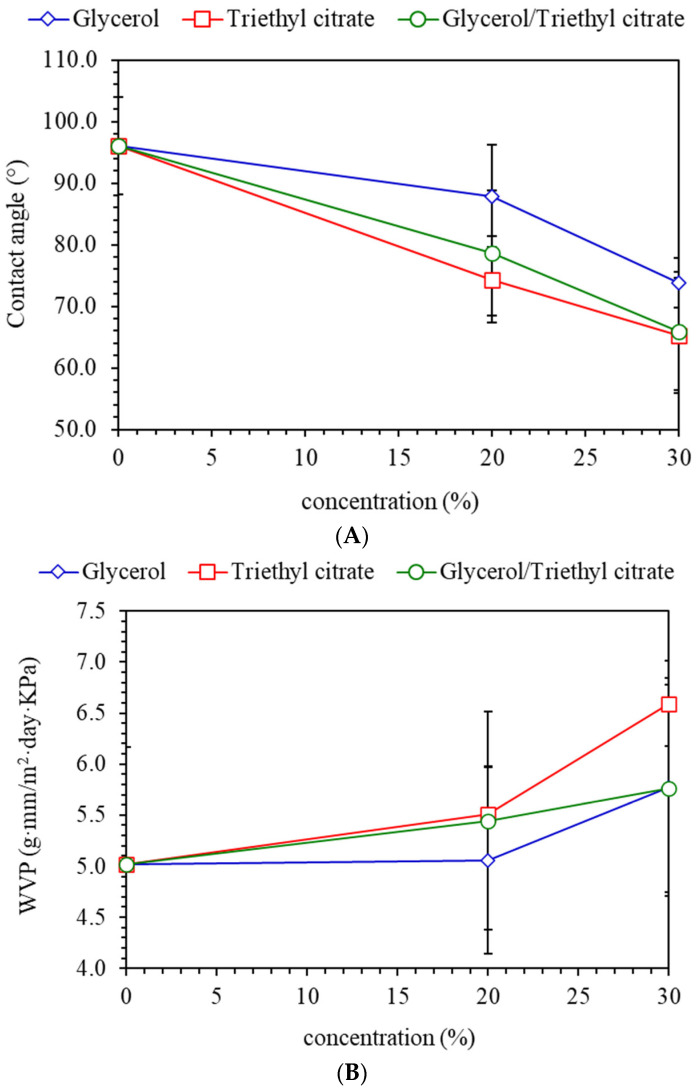

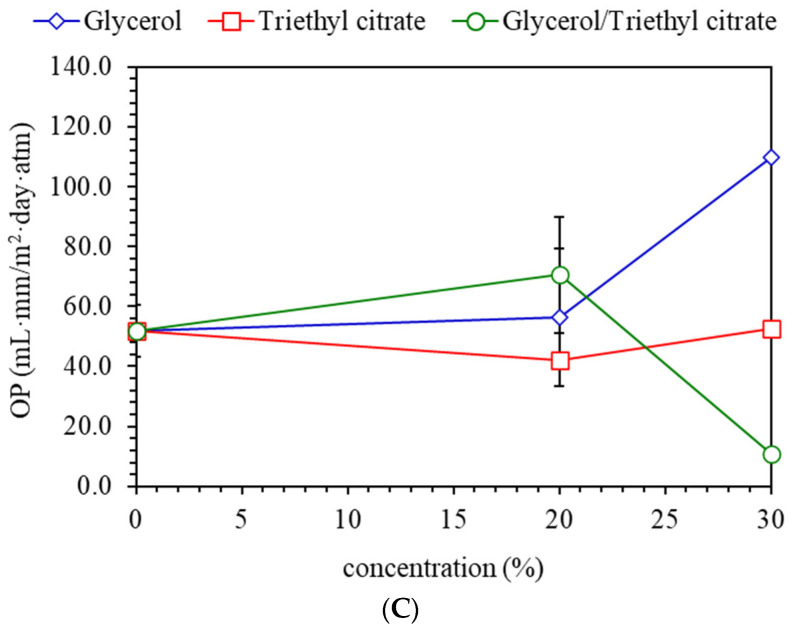
(**A**) Contact angle, (**B**) water vapor permeability (WVP) and (**C**) oxygen permeability (OP) of *Ulva rigida* films containing glycerol, triethyl citrate and their blends (glycerol/triethyl citrate) at 20% and 30%.

Surface hydrophobicity was determined according to water contact angle (CA), as shown in Figure 7A. CA of the control film was 96°, which is considered a hydrophobic surface (CA > 90°) [11]. Adding plasticizers clearly decreased CA, indicating the decreasing hydrophobicity of the film surface. Plasticization with glycerol greatly enhanced the hydrophilicity of the matrices, as reflected by higher degree of water absorption; however, CA was higher than CA of films containing triethyl citrate. Lower CA values of triethyl citrate-plasticized films reflected higher surface energy. The wettability of films depends on surface energy and surface roughness. A lower surface roughness reduced hydrophobic surface wettability [14,26,27]. The results suggested that adding triethyl citrate reduced the surface roughness of the films, corresponding with lower CA values. Plasticizer mixtures gave intermediate CA values between glycerol and triethyl citrate. Accordingly, the wettability of seaweed films mainly depended on surface roughness.

Water vapor permeability (WVP) and oxygen permeability (OP) are important parameters in food packaging that affect the quality and stability of packaged products. The WVP changed insignificantly when adding 20% plasticizers, while further increasing the plasticizer content increased WVP (Figure 7B). The hydroxyl groups and oxygen atoms in ester structures of citrate readily absorbed water, increasing diffusion of water vapor through the matrices [28]. However, plasticizers also decreased T_g_, which increased the molecular mobility of the matrices and crystallization. The formation of ordered and tightly packed crystalline structures inhibited the diffusion of volatile molecules including water vapor and gas [25,29]. Accordingly, the WVP values of the films insignificantly increased with the addition of 20% plasticizers. However, increasing the plasticizer content to 30% enhanced mobility and diffusion, which increased WVP. Similarly, adding 20% plasticizers led to insignificant changes in OP (Figure 7C). Increasing plasticizers also increased diffusion rates through the polymers due to increasing molecular mobility. However, blending plasticizers at 30% reduced OP due to high crystallinity which inhibited oxygen diffusion.

### 3.8. Tensile Properties 

**Figure 8:**
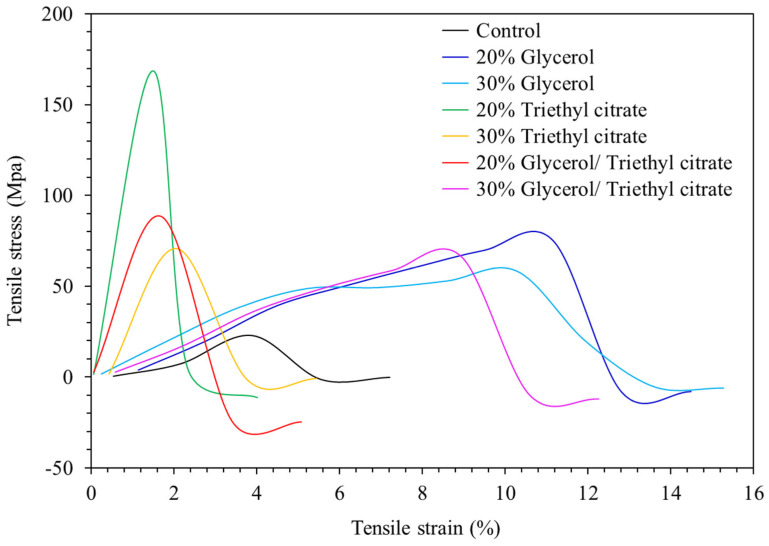
Tensile stress–strain curves of *Ulva rigida* films containing glycerol, triethyl citrate and their blends (glycerol/triethyl citrate) at 20% and 30%.

Mechanical properties were determined as tensile stress and tensile strain curves (Figure 8). The control film was weak and least flexible due to low plasticization effects. Polysaccharides consist of numerous hydroxyl groups that form strong inter- and intra-molecular hydrogen bonding. Consequently, the networks were rigid and required plasticization to improve deformability [13,25]. The extract consisted of small-molecular-weight amino acids, particularly aspartic, glutamic, alanine and glycine, which plasticized the matrices (Table 1). However, the results clearly demonstrated that plasticization efficiency was not sufficient to form flexible networks, and higher plasticizer contents were required.

Figure 8 shows that adding glycerol and triethyl citrate significantly improved tensile stress. Glycerol clearly increased tensile strain, reflecting the enhanced elongation of the films by up to 3-fold, while tensile stress was enhanced by up to 4-fold. Adding triethyl citrate at 20% greatly enhanced tensile stress but reduced strain values, indicating lower deformability and higher rigidity. Triethyl citrate induced crystallization of film components. The formation of immiscible rigid particles in the matrices caused non-homogeneous polymer networks, as also shown by SEM (Figure 2). These rigid particles acted as reinforcement, improving tensile stress by up to 8.5-fold (films with 20% triethyl citrate). However, the non-homogeneity decreased the area for distribution of applied external stress, leading to a lower extension ability [30]. Increasing triethyl citrate to 30% reduced strength because the large rigid particles increased non-homogeneity and decreased adhesion between the polymer networks [8,14]. Blending of plasticizers showed no synergistic improvement in mechanical strength. Glycerol (20–30%) greatly improved tensile properties and produced flexible seaweed films.

## 4. Conclusions

The water-soluble fraction of *Ulva rigida* consisted of polysaccharides and amino acid mixtures. Major amino acids were aspartic acid, glutamic acid, alanine and glycine, while polysaccharide structures consisted of rhamnose and sulfate derivatives. Native *Ulva rigida* extracts can form film networks; however, mechanical properties were poor, with very limited extensivity. Adding plasticizers such as glycerol and triethyl citrate improved the mechanical properties. These plasticizers interacted with *Ulva rigida* components via hydrogen bonding and modified the crystal morphology. Glycerol showed greater plasticization effects, resulting in a higher reduction in glass transition temperature and higher tensile strain, indicated by improved film elongation by up to 3-fold. Triethyl citrate greatly improved tensile stress but limited elongation. Film barrier properties were unaltered at up to 20% plasticizers, while increasing plasticizers to 30% generally increased water vapor and oxygen permeability due to increasing molecular mobility. The plasticization of *Ulva rigida* polymers produced efficient bioplastic films as a novel bioresource for sustainable food packaging.

## Figures and Tables

**Table 1:** Amino acid composition of *Ulva rigida* seaweed extract as determined via HPLC.

Amino Acid Composition (mg/100 g)	
Aspartic acid	2055.87 ± 47.90
Glutamic acid	1433.68 ± 28.79
Serine	823.04 ± 19.28
Histidine	252.36 ± 5.08
Glycine	1034.79 ± 18.34
Threonine	531.57 ± 15.39
Arginine	769.26 ± 16.84
Alanine	1097.61 ± 25.23
Tyrosine	517.68 ± 6.31
Cystine	745.40 ± 16.80
Valine	345.38 ± 5.88
Methionine	290.81 ± 2.44
Phenylalanine	629.89 ± 16.22
Isoleucine	214.56 ± 2.47
Leucine	749.68 ± 19.07
Lysine	561.83 ± 13.86
Tryptophan	181.37 ± 3.51
Proline	670.66 ± 34.40

## Data Availability

The data that support the findings of this study are available on request from the corresponding author.

## Supplementary Materials

The following supporting information can be downloaded at: https://www.mdpi.com/article/10.3390/polym15163342/s1, Table S1. FTIR peaks and corresponding functional group; Table S2. TGA onset temperature, weight loss in each stage of degradation and char residue.
