# Supplementary material for: Preparation and Characterization of Novel Green Seaweed Films from Ulva rigida
Source: Polymers (Basel). 2023 Aug 8;15(16):3342. doi: 10.3390/polym15163342 (PMC10460084; doi:10.3390/polym15163342)
Supplement: Supplementary file 1 [file polymers-15-03342-s001.zip › polymers-2515727-supplementary.pdf]

Supplementary Table S1. FTIR peaks and corresponding functional group

Film	Functional group	Mode	Wavenumber (cm^{-1})
Control film	C-O	Stretching	1026<br>1055
	C-O-S	Asymmetrical sulphate stretching	980
	C-O-S	Symmetrical sulphate stretching	848
Addition of glycerol and triethyl citrate	C-H	Bending	1500–1200
	C-O	Stretching	980–1250
	C=O in ester bonds	Stretching	1735
	C-OH in O–C=O	Deforming and symmetric stretching	1425
	HO-C=O	Stretching	1629
	S=O	Stretching	1200–1250
	C–O–S	Stretching	840–845
	O-H	Stretching,intra- and inter-molecular hydrogen bonding	3000–3700
	C-H	Stretching	2850–3050

Supplementary Table S2. TGA onset temperature, weight loss in each stage of degradation and char residue

Sample	First stage		Second stage		Third stage		Fourth stage		Char residue (%)
	T_{onset}(°C)	Wt loss(%)	T_{onset}(°C)	Wt loss(%)	T_{onset}(°C)	Wt loss(%)	T_{onset}(°C)	Wt loss(%)	
Control	52.3	28.5	206.4	31.2	574.7	9.3	724.0	9.5	21.5
20% glycerol	52.2	32.3	206.7	39.2	574.8	9.7	724.8	7.1	11.7
30% glycerol	52.2	33.1	206.7	40.9	574.7	9.2	724.8	7.0	9.7
20% triethyl citrate	52.2	31.1	207.2	32.2	575.5	10.9	725.7	8.9	16.9
30% triethyl citrate	52.2	30.3	207.7	32.3	575.5	11.4	725.7	8.8	17.2
20% glycerol/ triethyl citrate	51.7	31.4	207.7	35.7	575.0	10.0	724.8	8.7	14.2
30% glycerol/ triethyl citrate	52.2	31.0	207.2	39.9	574.7	10.7	725.3	7.8	10.6
